# Lanthanide complexes with d-f transition: new emitters for single-emitting-layer white organic light-emitting diodes

**DOI:** 10.1038/s41377-023-01211-5

**Published:** 2023-07-07

**Authors:** Peiyu Fang, Peihao Huo, Liding Wang, Zifeng Zhao, Gang Yu, Yanyi Huang, Zuqiang Bian, Zhiwei Liu

**Affiliations:** Beijing National Laboratory for Molecular Sciences, State Key Laboratory of Rare Earth Materials Chemistry and Applications, College of Chemistry and Molecular Engineering, Peking University, 100871 Beijing, China

**Keywords:** Organic LEDs, Photonic devices

## Abstract

White organic light-emitting diodes (WOLEDs) is a new generation of lighting technology and has stimulated wide-ranging studies. Despite the advantage of simple device structure, single-emitting-layer WOLEDs (SEL-WOLEDs) still face the challenges of difficult material screening and fine energy level regulation. Herein, we report efficient SEL-WOLEDs with a sky-blue emitting cerium(III) complex Ce-TBO^2Et^ and an orange-red emitting europium(II) complex Eu(Tp^2Et^)_2_ as the emitters, showing a maximum external quantum efficiency of 15.9% and Commission Internationale de l’Eclairage coordinates of (0.33, 0.39) at various luminances. Most importantly, the electroluminescence mechanism of direct hole capture and hindered energy transfer between the two emitters facilitate a manageable weight doping concentration of 5% for Eu(Tp^2Et^)_2_, avoiding the low concentration (<1%) of the low-energy emitter in typical SEL-WOLEDs. Our results indicate that d-f transition emitters may circumvent fine energy level regulation and provide development potential for SEL-WOLEDs.

## Abstract (web summary)

Luminescent lanthanide cerium(III) and europium(II) complexes with d-f transition characteristic were demonstrated as new emitters for efficient and color-stable single-emitting-layer white organic light-emitting diodes.

## Graphical abstract

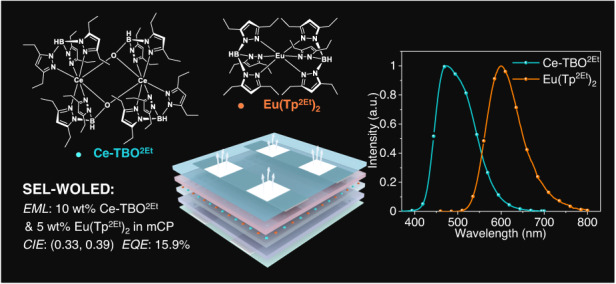

## Introduction

White organic light-emitting diodes (WOLEDs) have a great application prospect in daily lighting owing to their merits of low energy consumption, eye protection, and additional flexibility potential^1–6^. Compared with stacked WOLEDs^7^ or multiple-emitting-layer WOLEDs^8,9^, single-emitting-layer WOLEDs (SEL-WOLEDs) are favored for commercialization due to the drastically simplified device structure and reduced production costs^10,11^. However, a formidable challenge lies in the rational control of both singlet and triplet excitons between host materials and different color emitters in the single-emitting-layer^12–16^, making simultaneous efficiency improvement and color control a fundamental issue in SEL-WOLEDs^17–22^. Recently, considerable efforts have been devoted to molecular design and energy level regulation in host-guest multicomponent molecular systems^22–27^.

The observed white electroluminescence in typical SEL-WOLEDs usually involves complicated energy transfer processes from host material to emitters and between different color emitters. Even for the relatively simple binary system with blue and yellow/red emitters in the single-emitting-layer, the Förster resonance energy transfer, Dexter energy transfer, and the direct charge capture by luminescent materials all together induce the device design complexity^28–30^. Furthermore, efficient energy transfer between different color emitters makes the emission spectra correlate greatly to the doping concentration. Actually, rather low doping concentration (<1%) of the low-energy emitting material is generally needed^22,31–34^, which further increases the device fabrication difficulty.

In order to simplify device design and fabrication, reducing energy transfer channels between host material and emitters in the multicomponent single-emitting-layer is an intuitive method. Recently, we found that host materials are hardly involved in energy transfer process when luminescent lanthanide d-f transition complexes were used as the emitters^35,36^, which may be a solution for simple energy transfer regulation. In addition, we have demonstrated that d-f transition complexes have many advantages as emitters in OLEDs, such as theoretical high efficiency, short excited state lifetime, tunable emission color, and inexpensive cost due to the abundance of cerium in Earth’s crust is even slightly higher than that of copper^35–40^. Therefore, we could expect the exploration of d-f transition emitters in SEL-WOLEDs.

As a proof of concept, we synthesized a sky-blue emitting cerium(III) complex Ce-TBO^2Et^ and an orange-red emitting europium(II) complex Eu(Tp^2Et^)_2_ with d-f transition characteristic, and fabricated their SEL-WOLEDs with a simple three-layered device structure. It is found that the energy transfer from host material to emitters is eliminated and that between two emitters is hindered, hence we obtained efficient and color-stable white electroluminescence with a controllable weight doping ratio of 10% Ce-TBO^2Et^ and 5% Eu(Tp^2Et^)_2_ in *N*,*N*-dicarbazolyl-3,5-benzene (mCP).

## Results

### Synthesis and structure

**Fig. 1:**
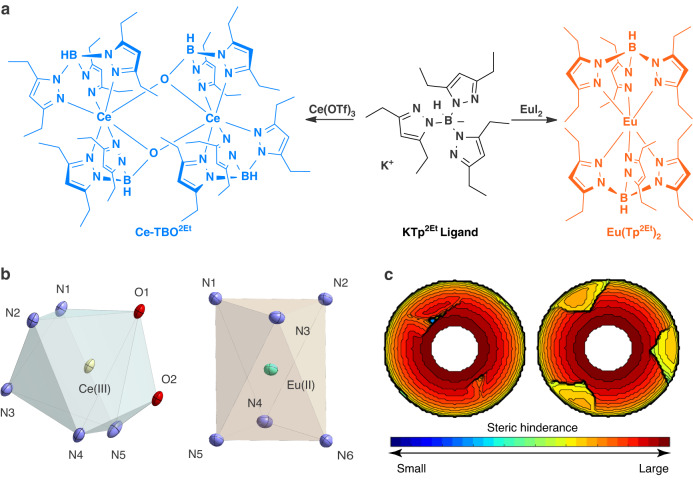
Synthesis and structural characterizations. **a** Synthesis and structures of Ce-TBO^2Et^ and Eu(Tp^2Et^)_2_. **b** Coordination geometry of Ce-TBO^2Et^ (left) and Eu(Tp^2Et^)_2_ (right). Atom notation: Ce, yellow; Eu, cyan; N, blue; O, red. **c** Steric maps of Ce-TBO^2Et^ (left) and Eu(Tp^2Et^)_2_ (right), hydrogen atoms are considered in the calculation, parameters adopted here are Bondi radii scaled by 1.17, sphere radius 3.5 Å, and mesh spacing value *s* = 0.10 Å

Based on the previous work^38,39,41^, potassium hydrotris(3,5-diethylpyrazolyl)borate (KTp^2Et^)^42^ is chosen to synthesize luminescent lanthanide d-f transition complexes due to its rigid coordination skeleton and good protection for the metal center, as well as our prediction that the moderate ligand field would lead to sky-blue emitting Ce(III) complex Ce-TBO^2Et^ and orange-red emitting Eu(II) complex Eu(Tp^2Et^)_2_ as depicted in Fig. 1a. The complexes were characterized by elemental analysis and X-ray single crystal diffraction (Supplementary Table S1). Due to the large steric hinderance of two ethyl groups in the Tp^2Et^ ligand which weakens the B–N bond^43^, oxygen is easily introduced in Ce-TBO^2Et^ as a bridging coordination atom by the addition of water in the reaction mixture. Thus the Ce(III) ion in Ce-TBO^2Et^ is coordinated with five N atoms and two O atoms (Supplementary Fig. S1), resulting in a distorted single-capped-octahedral coordination geometry as depicted in Fig. 1b, and the average Ce−N and Ce−O bond distances are 2.609 Å and 2.333 Å, respectively. In the complex Eu(Tp^2Et^)_2_, the central Eu(II) ion is coordinated with six N atoms and encapsulated in a staggered manner with a trigonal antiprismatic coordination geometry (Fig. 1b, Supplementary Fig. S1), and the average Eu−N bond distance is 2.611 Å. The percentages of buried volume (%*V*_Bur_)^44^, which measures the compactness of the first coordination sphere, are calculated to be 92% for Ce-TBO^2Et^ and 83% for Eu(Tp^2Et^)_2_, and the steric maps are shown in Fig. 1c.

### Photophysical properties

**Fig. 2:**
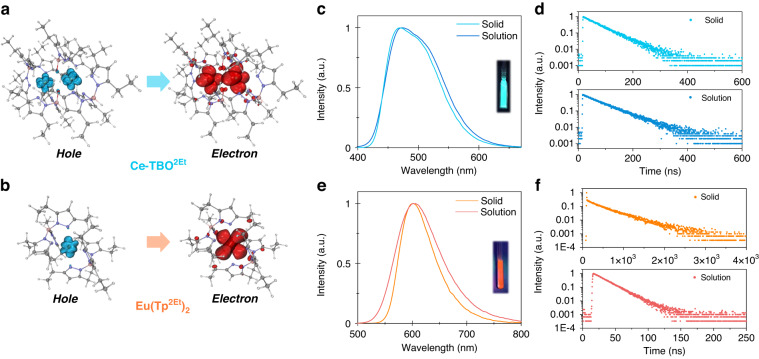
TD-DFT calculation results and photophysical properties. **a** Distributions of the hole and electron for the lowest energy transition (at 407 nm) for Ce-TBO^2Et^. **b** Distributions of the hole and electron for the lowest energy transition (at 423 nm) for Eu(Tp^2Et^)_2_. **c** Emission spectra of Ce-TBO^2Et^, inset is the photograph of solution under 365 nm radiation. **d** Transient photoluminescence decay curves of Ce-TBO^2Et^. **e** Emission spectra of Eu(Tp^2Et^)_2_, inset is the photograph of solution under 365 nm radiation. **f** Transient photoluminescence decay curves of Eu(Tp^2Et^)_2_. Solid represents the complex in solid powder state. Solution represents the complex in 1 mM dichloromethane solution

To systematically study the photophysical properties of the two complexes, steady-state and transient spectra were measured in dichloromethane solution as well as in solid powder state under nitrogen atmosphere. All the photoluminescence data are summarized in Table 1. When dispersed in dichloromethane solution, the broad and featureless absorption bands above 300 nm were detected, which are attributed to the 4f^1^ → 5d^1^ transition of Ce(III) ion and 4f^7^ → 4f^6^5d^1^ transition of Eu(II) ion, respectively (Supplementary Fig. S2). Further time-dependent density functional theory (TD-DFT) calculation with hole-electron analysis (Fig. 2a, b) and natural transition orbital (NTO) analysis (Supplementary Fig. S3) confirmed the dominant f-d transition characteristics, and the predicted absorption bands are consistent with the experimental data (Table 1). The photoluminescence spectra of Ce-TBO^2Et^ in dichloromethane solution and as solid powder both displayed sky-blue emissions with maximum emission wavelengths (*λ*_max_) of 469 nm and 474 nm, respectively (Fig. 2c). The short single-exponential fluorescence decay lifetimes (*τ*) of 66 ns and 55 ns for Ce-TBO^2Et^ in dichloromethane solution and as solid powder (Fig. 2d) coincide with general lifetime span of d-f transition Ce(III) complexes^35,45^. Moreover, Ce-TBO^2Et^ exhibited high photoluminescence quantum yield (PLQY, *Φ*_PL_) close to 100% both in dichloromethane solution and as solid powder (Table 1). The radiative rate constants (*k*_r_) and non-radiative rate constants (*k*_nr_) of Ce-TBO^2Et^ were calculated. The complex in dichloromethane solution and as solid powder showed similar high *k*_r_ (1.8 × 10^7 ^s^−1^ *vs*. 1.5 × 10^7 ^s^−1^) as well as similar low *k*_nr_ (0.018 × 10^7 ^s^−1^ *vs*. 0.015 × 10^7 ^s^−1^), indicating a rigid coordination of the ligands to the central Ce(III) ion.

**Table 1:** The summary of photophysical properties of Ce-TBO^2Et^ and Eu(Tp^2Et^)_2_

Complex	Ce-TBO^2Et^			Eu(Tp^2Et^)_2_		
State	Solid	Solution^a^	Film^b^	Solid	Solution^a^	Film^b^
*λ*_abs_ [nm]^c^	–	398 (407)	397	–	403 (423)	405
*λ*_em_ [nm]^d^	469	474	475	600	604	593
*Φ*_PL_ [%]^e^	~100	~100	98	20	2	28
*τ* [ns]^f^	55	66	60	428	17	110, 632
*k*_r_ [10^7 ^s^−1^]^g^	1.8	1.5	1.6	0.047	0.12	0.049^i^
*k*_nr_ [10^7 ^s^−1^]^h^	0.018	0.015	0.033	0.19	5.8	0.12^i^

^a^Measured in dichloromethane solution (1 mM)

^b^PMMA film with the doping concentration of 10 wt%

^c^Experimental absorption peaks and the TD-TDFT calculated results are given in parentheses

^d^Maximum emission wavelength

^e^PLQY

^f^Excited state lifetime

^g^*k*_r_ is obtained from equation *k*_r_ = *Φ*_PL_/*τ*

^h^*k*_nr_ is obtained from equation *k*_nr_ = (1 − Φ _PL_)/*τ*

^i^Calculated with average excited state lifetime of 574 ns

The photoluminescence spectra of Eu(Tp^2Et^)_2_ in dichloromethane solution and as solid powder both displayed orange-red emissions with maximum wavelengths of 600 nm and 604 nm (Fig. 2e), and the excited state lifetimes are 17 ns and 428 ns (Fig. 2f), respectively. The single-peak spectra and nanosecond excited state lifetimes of Eu(Tp^2Et^)_2_ are consistent with those of the reported d-f transition Eu(II) complexes^37,38^. In addition, the *Φ*_PL_ values of Eu(Tp^2Et^)_2_ in dichloromethane solution and as solid powder are only 2% and 20%, respectively. The *k*_r_ and *k*_nr_ of Eu(Tp^2Et^)_2_ in dichloromethane solution and as solid powder were also calculated, showing much higher *k*_nr_ than *k*_r_. However, the complex exhibited lower *k*_nr_ in solid powder state than that in solution, indicating that non-radiative channels are suppressed due to the more rigid environment in solid state, therefore, the *Φ*_PL_ in solid powder is greatly improved. Compared with Ce-TBO^2Et^, the *λ*_max_ of Eu(Tp^2Et^)_2_ is red shifted and the *Φ*_PL_ is decreased. The former may arise mainly from higher energy *E*_fd_ for the transition between the lowest 4f^n^5d^0^ and the lowest 4f^n−1^5d^1^ states of free (gaseous) Ce(III) ion than that of Eu(II) ion (~6 eV *vs*. ~4 eV)^46^, while the latter may be the result of lower encapsulation of Eu(II) ion by Tp^2Et^ ligands, i.e. smaller %*V*_Bur_ as calculated above (92% for Ce-TBO^2Et^ and 83% for Eu(Tp^2Et^)_2_), leading to the nonnegligible quenching of luminescence.

We have also respectively doped Ce-TBO^2Et^ and Eu(Tp^2Et^)_2_ in poly(methylmethacrylate) (PMMA) film with a doping concentration of 10 wt%. The photophysical properties of the doped films under nitrogen atmosphere are depicted in Supplementary Fig. S4 and summed in Table 1. The absorption spectra are consistent with that of dichloromethane solution (Supplementary Figs. S4a and S2). The emission peaks of Ce-TBO^2Et^ and Eu(Tp^2Et^)_2_ doped in PMMA films are 475 nm and 593 nm (Supplementary Fig. S4b), respectively. The excited state lifetime of Ce-TBO^2Et^ doped in PMMA film is 60 ns, comparable to that of solid powder and solution, and the *Φ*_PL_ is 98% (Table 1). When doped in PMMA film, Eu(Tp^2Et^)_2_ exhibits longer excited state lifetimes (110 ns and 632 ns, Supplementary Fig. S4c) as well as a higher *Φ*_PL_ of 28% compared to that of solid powder and solution (Table 1), which may result from the suppressed concentration and solvent quenching.

### Thermal and electrochemical properties

During the synthesis, we found that both Ce-TBO^2Et^ and Eu(Tp^2Et^)_2_ can be purified by thermal gradient sublimation, making them suitable candidates for OLEDs fabrication with the vacuum thermal evaporation method. The detailed thermal stabilities of Ce-TBO^2Et^ and Eu(Tp^2Et^)_2_ were examined by thermogravimetric analysis and differential scanning calorimetry under nitrogen atmosphere (Supplementary Fig. S5). The complexes exhibited high decomposition temperatures (*T*_d_, corresponding to 5% weight loss) above 240 °C, high glass-transition temperature (*T*_g_, 139 °C for Ce-TBO^2Et^) and high melting temperatures (*T*_m_) above 160 °C, indicating good thermal stability for OLEDs application.

To understand the frontier energy levels of the two complexes, electrochemical properties of Ce-TBO^2Et^ and Eu(Tp^2Et^)_2_ were measured by cyclic voltammogram (Supplementary Fig. S6). The oxidation onsets against ferrocenium/ferrocene (Fc^+^/Fc) were observed as 1.0 V and −0.6 V for Ce-TBO^2Et^ and Eu(Tp^2Et^)_2_, respectively. Thus, the highest occupied molecular orbital (HOMO) energy levels are deduced to be −5.8 eV and −4.2 eV, respectively. This leads us to the conclusion that Eu(Tp^2Et^)_2_ has a much shallower HOMO energy level than that of Ce-TBO^2Et^. The lowest unoccupied molecular orbital (LUMO) energy levels were then calculated to be −3.0 eV and −1.9 eV for Ce-TBO^2Et^ and Eu(Tp^2Et^)_2_, by considering the HOMO-LUMO energy gaps of 2.8 eV and 2.3 eV predicted from the absorption spectra, respectively (Supplementary Fig. S2).

### Electroluminescence performance

Due to the important role of host material in OLEDs, serval host materials were evaluated by measuring the *Φ*_PL_ of Ce-TBO^2Et^ or Eu(Tp^2Et^)_2_ doped films. In particular, the *Φ*_PL_ of Ce-TBO^2Et^ doped in mCP film could be as high as ~100%, and the *Φ*_PL_ of Eu(Tp^2Et^)_2_ doped in *N*,*N*’-di-[(1-naphthalenyl)-*N*,*N*’-diphenyl]-1,1’-biphenyl)-4,4’-diamine (NPB), 4,4’,4”-tris(*N*-3-methylphenyl-*N*-phenylamino)triphenylamine (m-MTDATA), and mCP films are 46%, 46%, and 57%, respectively (Supplementary Table S2, Supplementary Fig. S7). Thus, mCP is considered as a good host material for both Ce-TBO^2Et^ and Eu(Tp^2Et^)_2_, and used as host material in the fabrication of the following OLEDs. It should be noted that the *Φ*_PL_ of Eu(Tp^2Et^)_2_ doped in mCP film increased significantly when compared with that in solution or in solid powder state, indicating that solvent quenching and concentration quenching may be suppressed in the doped film.

**Fig. 3:**
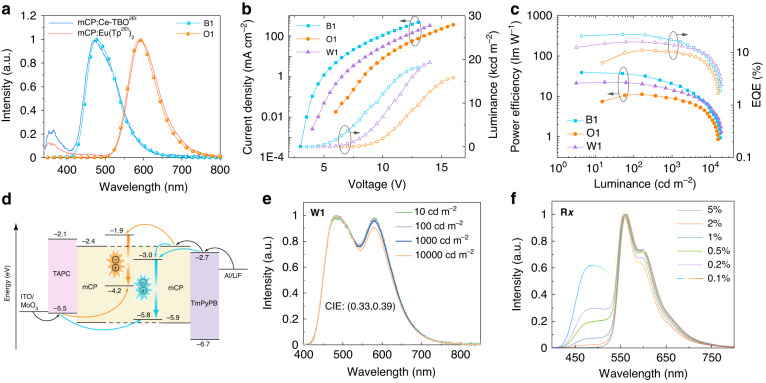
Properties and mechanism of electroluminescent devices. **a** Photoluminescence spectra of 10 wt% Ce-TBO^2Et^ doped mCP film and 12 wt% Eu(Tp^2Et^)_2_ doped mCP film (the excitation wavelength is 270 nm and 300 nm, respectively), and electroluminescence spectra of devices B1 and O1. **b** Current density-voltage-luminance traces of the devices B1, O1, and W1. **c** Power efficiency-luminance-EQE traces of the devices B1, O1 and W1. **d** Schematic device configuration and electroluminescence mechanism diagram of devices B1, O1 and W1. **e** Electroluminescence spectra of the device W1 at different luminances. **f** Electroluminescence spectra of devices R*x* with different doping concentrations of Ir(bt)_2_acac

Prior to constructing SEL-WOLEDs, the electroluminescence properties of Ce-TBO^2Et^ as a sky-blue emitter and Eu(Tp^2Et^)_2_ as an orange-red emitter were separately investigated by preparing the blue emitting device B1 and the orange-red emitting device O1 with a same device structure of indium tin oxide (ITO)/MoO_3_ (2 nm)/1-bis[4-[*N*,*N*’-di(4-tolyl)amino]phenyl]cyclohexane (TAPC, 60 nm)/mCP:Ce-TBO^2Et^ (10 wt%, 30 nm) or mCP:Eu(Tp^2Et^)_2_ (12 wt%, 25 nm)/1,3,5-tri(m-pyrid-3-yl-phenyl)benzene (TmPyPB, 40 nm)/LiF (0.7 nm)/Al (100 nm). In the devices, MoO_3_ and TAPC serve as the hole injection layer and the hole transport layer, TmPyPB and LiF serve as the electron transport layer and the electron injection layer, respectively (see Supplementary Fig. S7 for the chemical structures of TAPC and TmPyPB). The two devices B1 and O1 showed only electroluminescence of emitters and no emission of host material was observed, which are different from the photoluminescence spectra of the emitting-layers with simultaneous emissions of the complex and mCP, respectively (Fig. 3a). The Ce-TBO^2Et^ based device B1 showed a maximum luminance of 18,200 cd m^−2^ and a maximum EQE of 22.3%, while the Eu(Tp^2Et^)_2_ based device O1 showed a maximum luminance of 15,800 cd m^−2^ and a maximum EQE of 11.1% (Table 2, Fig. 3b, c). Noticeably, these performance surpass the best reported blue and red OLEDs with d-f transition lanthanide complexes (Supplementary Table S3)^35–41,45,47,48^.

**Table 2:** Electroluminescence performance of the fabricated OLEDs

Device	*V* [V]^a^	*L*_max_ [cd m^−2^]^b^	*η*_CE_ [cd A^−1^]^c^	*η*_EQE_ [%]^d^	CIE^e^
B1	3.2/5.6/9.5	18,200	47.0/33.9/12.6	22.3/16.1/6.0	(0.18, 0.32)
O1	6.3/9.4/13.2	15,800	28.7/28.7/11.0	11.1/10.2/4.3	(0.54, 0.46)
W1	4.2/7.4/10.8	19,300	38.4/33.1/17.7	15.9/13.8/7.4	(0.33, 0.39)

^a^Driving voltage at 1, 1000 and 10,000 cd m^−2^

^b^Maximum luminance

^c^Maximum current efficiency and current efficiencies at 1000 and 10,000 cd m^−2^

^d^Maximum EQE and EQEs at 1000 and 10,000 cd m^−2^

^e^CIE at 10,000 cd m^−2^

The differences between electroluminescence and photoluminescence spectra of Ce-TBO^2Et^ or Eu(Tp^2Et^)_2_ in mCP suggest that hole and electron recombination dominantly occurs on d-f transition complexes rather than the host material, which avoids energy transfer from host molecule to the doped complex, so the emission of host material is no longer observed in the electroluminescence spectra. To clarify the electroluminescence mechanism, we fabricated two sets of hole-only devices with a device structure of ITO/MoO_3_ (2 nm)/mCP (40 nm)/mCP:Ce-TBO^2Et^ or Eu(Tp^2Et^)_2_ (0, 5, 10 or 15 wt%, 25 nm)/mCP (40 nm)/MoO_3_ (2 nm)/Al. It is found that the current density of Ce-TBO^2Et^ and Eu(Tp^2Et^)_2_ devices both reduced significantly first and raised then with the increased doping concentration of the complex, indicating that Ce-TBO^2Et^ and Eu(Tp^2Et^)_2_ have the capability to capture holes (Supplementary Fig. S8). Furthermore, we could infer that Eu(Tp^2Et^)_2_ has priority to capture holes due to a much shallower HOMO energy level compared with that of Ce-TBO^2Et^. It is worth mentioning that although the excitons are not formed on mCP, the host material is important for electron transport. Furthermore, the high-energy triplet state (2.9 eV) of mCP is essential to prevent energy transfer from dopant to host material. Based on aforementioned studies, the electroluminescence mechanism of Ce-TBO^2Et^ and Eu(Tp^2Et^)_2_ could be depicted in Fig. 3d. The holes transported by TAPC were captured by Ce-TBO^2Et^ and Eu(Tp^2Et^)_2_, and then recombined with electrons transported by TmPyPB and mCP.

Encouraged by the results that both efficient sky-blue electroluminescence and orange-red electroluminescence were realized with the same simple three-layered device structure, we designed a SEL-WOLED by only changing the single emitting-layer with Ce-TBO^2Et^ and Eu(Tp^2Et^)_2_ co-doped in mCP, and fabricated the device W1: ITO/MoO_3_ (2 nm)/TAPC (60 nm)/mCP:Ce-TBO^2Et^ (10 wt%):Eu(Tp^2Et^)_2_ (5 wt%) (25 nm)/TmPyPB (40 nm)/LiF (0.7 nm)/Al (100 nm). The device showed a Commission Internationale de l´Eclairage (CIE) of (0.33, 0.39) at 1000 cd m^−2^, with emission wavelengths peaking at 490 nm and 580 nm (Fig. 3e), arising from Ce-TBO^2Et^ and Eu(Tp^2Et^)_2_, respectively. And good color stability with CIE variation within (±0.01, ±0.01) from 10 to 10,000 cd m^−2^ was observed (Fig. 3e), meaning that the exciton allocations are basically independent on different exciton concentrations. The color temperature and color rendering index for the device W1 at 1000 cd m^−2^ are 5380 K and 76, respectively. Moreover, an intermediate efficiency between that of the devices B1 and O1 was obtained in the device W1, showing a maximum EQE of 15.9%, and the EQE is still up to 13.8% at a high luminance of 1000 cd m^−2^ (Fig. 3b, c, Table 2).

It is noticeable that the weight doping concentration of 5% for Eu(Tp^2Et^)_2_ is controllable, avoiding the low concentration (<1%) of the low-energy emitter in typical SEL-WOLEDs. To clarify the exceptionality, a series of reference devices R*x* using a classic phosphorescence iridium complex bis(2-phenylbenzothiazolato)(acetylacetonate)iridium(III) (Ir(bt)_2_acac)^49^ as the orange-red emitter were fabricated with a device structure of ITO/MoO_3_ (2 nm)/TAPC (60 nm)/mCP:Ce-TBO^2Et^ (10 wt%):Ir(bt)_2_acac (*x* wt%) (25 nm)/TmPyPB (40 nm)/LiF (0.7 nm)/Al (100 nm). It is found that the device with 5 wt% Ir(bt)_2_acac showed pure emission from Ir(bt)_2_acac, and no emission from Ce-TBO^2Et^ was observed (Fig. 3f). A comparable emission from the sky-blue emitter Ce-TBO^2Et^ to that of Ir(bt)_2_acac was measured until the doping concentration of Ir(bt)_2_acac is decreased to 0.1 wt%, resulting in a warm white emission with CIE coordinates of (0.33, 0.41) (Fig. 3f). The different optimal doping concentration of Eu(Tp^2Et^)_2_ in the device W1 compared with that of Ir(bt)_2_acac in the device R*x* (5% *vs*. 0.1%) indicates there may be distinctive energy transfer between Ce-TBO^2Et^ and Eu(Tp^2Et^)_2_.

**Fig. 4:**
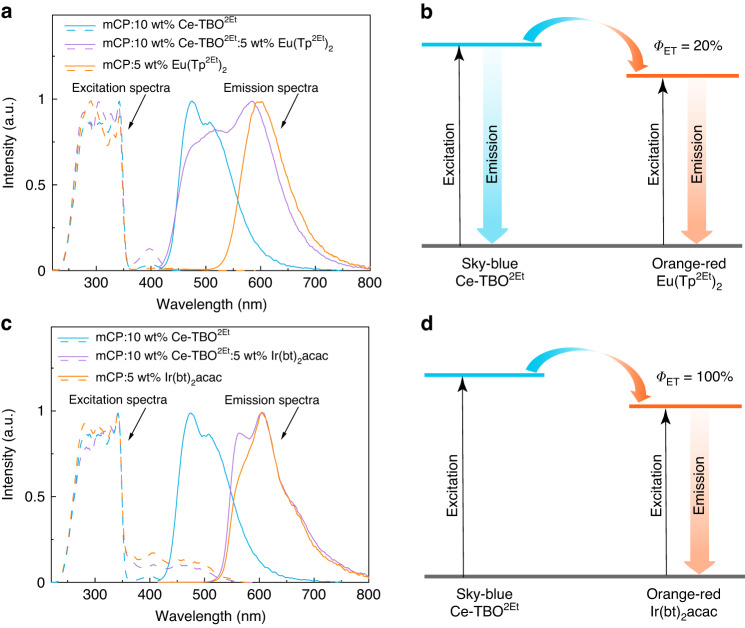
Photophysical properties of doped films and schematic diagrams of energy transfer mechanisms. **a** Excitation spectra (dashed lines) and emission spectra (solid lines) of mCP:10 wt% Ce-TBO^2Et^ (blue lines), mCP:5 wt% Eu(Tp^2Et^)_2_ (orange lines), and mCP:10 wt% Ce-TBO^2Et^:5 wt% Eu(Tp^2Et^)_2_ (purple lines) films. **b** Energy diagram of the co-doped mCP:10 wt% Ce-TBO^2Et^:5 wt% Eu(Tp^2Et^)_2_ film. **c** Excitation spectra (dashed lines) and emission spectra (solid lines) of mCP:10 wt% Ce-TBO^2Et^ (blue lines), mCP:5 wt% Ir(bt)_2_acac (orange lines), and mCP:10 wt% Ce-TBO^2Et^:5 wt% Ir(bt)_2_acac (purple lines) films. **d** Energy diagram of the co-doped mCP:10 wt% Ce-TBO^2Et^:5 wt% Ir(bt)_2_acac film. The excitation spectra are monitored at the maximum emission peak and the emission spectra are monitored at an excitation wavelength of 400 nm. *Φ*_ET_ represents the energy transfer efficiency from Ce-TBO^2Et^ to Eu(Tp^2Et^)_2_ or Ir(bt)_2_acac

In order to understand the energy transfer between Ce-TBO^2Et^ and Eu(Tp^2Et^)_2_ in the single-emitting-layer, three doped films mCP:10 wt% Ce-TBO^2Et^, mCP:5 wt% Eu(Tp^2Et^)_2_, and mCP:10 wt% Ce-TBO^2Et^:5 wt% Eu(Tp^2Et^)_2_ with the same thickness (300 nm) were fabricated by vacuum thermal evaporation and their photophysical properties were measured (Fig. 4, Table 3). For the doped films mCP:10 wt% Ce-TBO^2Et^ and mCP:5 wt% Eu(Tp^2Et^)_2_, weak f-d excitation peaks were both observed around 400 nm (Fig. 4a) with an absorption intensity (*A*) of 0.035 and 0.024 (Table 3), respectively. For the co-doped film mCP:10 wt% Ce-TBO^2Et^:5 wt% Eu(Tp^2Et^)_2_, the absorption intensity is measured to be 0.064 (Table 3), close to the sum of the above two values, suggesting independent excitation of the two emitters. The co-doped film mCP:10 wt% Ce-TBO^2Et^:5 wt% Eu(Tp^2Et^)_2_ exhibited two emission peaks at 490 and 600 nm, which is similar to the electroluminescence spectrum of the device W1 (Figs. 4a and 3e). The ratio of sky-blue photons to orange-red photons (*r*) in the co-doped film mCP:10 wt% Ce-TBO^2Et^:5 wt% Eu(Tp^2Et^)_2_ is ~1.4 as deduced from the peak splitting fitting of the spectrum (Supplementary Fig. S9), where the sky-blue and orange-red photons originate from Ce-TBO^2Et^ and Eu(Tp^2Et^)_2_, respectively. Theoretically, *r* can be calculated by Eq. (1):

1 $$r=\frac{{A}_{{\rm{B}}}\times {\varPhi }_{{\rm{PL}},{\rm{B}}}\times (1-{\varPhi }_{{\rm{ET}}})}{{A}_{{\rm{B}}}\times {\varPhi }_{{\rm{PL}},{\rm{B}}}\times {\varPhi }_{{\rm{ET}}}+{A}_{{\rm{O}}}\times {\varPhi }_{{\rm{PL}},{\rm{O}}}}$$

where *A* is absorption intensity and *Φ*_ET_ represents the energy transfer efficiency from the sky-blue emitter to the orange-red emitter, and the subscript B refers to the sky-blue emitter and the subscript O refers to the orange-red emitter. Therefore, the calculated *Φ*_ET_ from Ce-TBO^2Et^ to Eu(Tp^2Et^)_2_ is around 20% (Table 3) and the energy diagram of the co-doped film mCP:10 wt% Ce-TBO^2Et^:5 wt% Eu(Tp^2Et^)_2_ is depicted in Fig. 4b. The energy transfer from Ce-TBO^2Et^ to Eu(Tp^2Et^)_2_ is mostly inhibited, which accounts for the high doping concentration of Eu(Tp^2Et^)_2_ to achieve white emission in the device W1. As references, another two doped films mCP:5 wt% Ir(bt)_2_acac and mCP:10 wt% Ce-TBO^2Et^:5 wt% Ir(bt)_2_acac were also fabricated and their photophysical properties were studied (Fig. 4, Table 3). Though the absorption intensity of film mCP:10 wt% Ce-TBO^2Et^ at 400 nm is non-ignorable as compared with that of the film mCP:5 wt% Ir(bt)_2_acac (0.035 *vs*. 0.126), the co-doped film mCP:10 wt% Ce-TBO^2Et^:5 wt% Ir(bt)_2_acac exhibited an emission spectrum that is basically consistent with that of the film mCP:5 wt% Ir(bt)_2_acac (Fig. 4c), indicating that the energy absorbed by Ce-TBO^2Et^ is efficiently transferred to Ir(bt)_2_acac, and the *Φ*_ET_ from Ce-TBO^2Et^ to Ir(bt)_2_acac is deduced to be nearly 100% (Table 3, Fig. 4d). Therefore, white emission can be obtained only when the doping concentration of Ir(bt)_2_acac is reduced to a very low level in the reference device R*x*. In order to explore the different *Φ*_ET_ between Ce-TBO^2Et^ and Eu(Tp^2Et^)_2_ or Ir(bt)_2_acac, we overlap the absorption spectrum of 10 wt% Eu(Tp^2Et^)_2_ or Ir(bt)_2_acac doped PMMA film with the normalized emission spectrum of 10 wt% Ce-TBO^2Et^ doped PMMA film (Supplementary Fig. S10), and the overlapping area of Ce-TBO^2Et^ and Eu(Tp^2Et^)_2_ is much smaller than that of Ce-TBO^2Et^ and Ir(bt)_2_acac. This means the emission energy of Ce-TBO^2Et^ is less efficiently absorbed by Eu(Tp^2Et^)_2_ than by Ir(bt)_2_acac. Therefore, the *Φ*_ET_ from Ce-TBO^2Et^ to Eu(Tp^2Et^)_2_ is much lower than that from Ce-TBO^2Et^ to Ir(bt)_2_acac.

**Table 3:** Photophysical properties of the doped films

Film	*A*^a^	*Φ*_PL_ [%]^b^	*λ*_em_ [nm]^c^	*r*^d^	*Φ*_ET_ [%]^e^
mCP:10 wt% Ce-TBO^2Et^	0.035	100	476		
mCP:5 wt% Eu(Tp^2Et^)_2_	0.024	54	600		
mCP:5 wt% Ir(bt)_2_acac	0.126	94	605		
mCP:10 wt% Ce-TBO^2Et^:5 wt% Eu(Tp^2Et^)_2_	0.064	44	490, 600	1.4	20
mCP:10 wt% Ce-TBO^2Et^:5 wt% Ir(bt)_2_acac	0.172	77	606	~0	~100

The excitation wavelength is 400 nm

^a^Absorption intensity provided by the PLQY measurement system

^b^PLQY

^c^Maximum emission wavelength

^d^The ratio of sky-blue photons to orange-red photons

^e^Energy transfer efficiency from Ce-TBO^2Et^ to Eu(Tp^2Et^)_2_ or Ir(bt)_2_acac

## Discussion

In summary, a sky-blue emitting Ce(III) complex Ce-TBO^2Et^ and an orange-red emitting Eu(II) complex Eu(Tp^2Et^)_2_ with effective d-f transition are synthesized and characterized. The maximum EQEs of the corresponding sky-blue OLEDs and orange-red OLEDs reached 22.3% and 11.1%, which are the highest record of d-f transition based blue and red OLEDs, respectively. Using the two complexes with complementary colors, we fabricated efficient and color-stable three-layered SEL-WOLEDs, showing a maximum EQE of 15.9% and CIE coordinates of (0.33, 0.39) at various luminances. It is found that excitons are directly formed on Ce-TBO^2Et^ and Eu(Tp^2Et^)_2_ due to their hole capture capability in electroluminescent process, avoiding the energy transfer from host material to dopants. Moreover, hindered energy transfer between the two emitters provides an advantage on doping concentration optimization, i.e. avoiding the low concentration (<1%) of the low-energy emitter in typical SEL-WOLEDs. This work demonstrates that the application of d-f transition complexes is expected to bring a new perspective on SEL-WOLEDs construction with simplified energy level regulation and manufacturing process.

## Materials and methods

### General

All chemical reagents used in the synthesis process were commercially available and used as received unless otherwise mentioned. TAPC (99.5%) and TmPyPB (99%) are purchased from Xi’an Polymer Light Technology Corporation, and mCP (99.5%) is purchased from Jilin Oled Material Technology Corporation. K(Tp^2Et^) was synthesized as reported^42^. Synthesis of the Ce(III) and Eu(II) complexes were conducted in glove box. Ir(bt)_2_acac (99%) is purchased from Luminescence Technology Corporation and further purified by vacumm sublimation. Elemental analysis were performed on a VARIO EL analyzer (GmbH, Hanau, Germany).

### Spectroscopic measurements

Single crystal X-ray diffraction data were measured on Rigaku XtaLAB PRO 007HF (Mo). UV absorption spectra were collected on a Shimadzu UV3600PLUS. Photoluminescence efficiencies and absorption intensity of doped films were measured using an absolute photoluminescence quantum yield measurement system on C9920-02, Hamamatsu Company. Steady-state and transient photoluminescence spectra were measured on an Edinburgh Analytical Instruments FLS980 spectrophotometer. All the compounds, no matter in solid powder or in solution state, were under inert atmosphere protection when carrying out photophysical characterizations.

### Thermal stability measurements

Thermal gravimetric analysis (TGA) was carried out on TA Instruments SDT Q600. Differential scanning calorimetry (DSC) was measured on TA Instruments Q2000.

### Cyclic voltammetry (CV) measurements

CV was carried out under inert atmosphere protection in dichloromethane (Ce-TBO^2Et^) or hexane (Eu(Tp^2Et^)_2_) solution at room temperature with a CHI voltammetric analyzer. Tetrabutylammonium hexafluorophosphate (TBAPF_6_, 0.1 M) was used as the supporting electrolyte. The conventional three-electrode configuration consists of a platinum working electrode, a platinum wire auxiliary electrode, and an Ag/AgCl wire pseudo-reference electrode with ferrocene as the external standard. Cyclic voltammogram was obtained at a scan rate of 100 mV s^−1^.

### OLEDs fabrication and measurements

Indium tin oxide (ITO) patterned anode was commercially available with a sheet resistance of 14 Ω square^−1^ and a thickness of 80 nm. ITO substrates were cleaned with deionized water and ethanol. The organic and metal layers were deposited in different vacuum chambers with a base pressure better than 1 × 10^−4^ Pa. The active area for each device is 4 mm^2^. All electric testing and optical measurements were performed under ambient conditions with encapsulation of devices in a glove box. The electroluminescence spectra, current density-voltage-luminance curves, and EQE characteristics were measured by computer controlled Keithley 2400 source meter and absolute EQE measurement system (C9920-12) with photonic multichannel analyzer (PMA-12, Hamamatsu Photonics).

### Synthesis of Ce-TBO^2Et^

Ce(CF_3_SO_3_)_3_ (5.87 g, 10.0 mmol), KTp^2Et^ (8.41 g, 20.0 mmol), and H_2_O (0.180 g, 10.0 mmol) were added to a 100 mL round-bottomed flask with 50 mL tetrahydrofuran in a glove box. The mixture was stirred for 3 days at room temperature and then the solvent was removed under vacuum. The mixture was washed with hexane to obtain pale yellow solid powder. Pure product was obtained as crystalline powder after sublimation of the yield solid powder at 250 °C (0.560 g, 0.352 mmol, 7%). Anal. calcd for C_70_H_114_B_4_N_20_O_2_Ce_2_: C 52.84, H 7.22, N 17.60; found: C 52.97, H 7.34, N 17.82.

### Synthesis of Eu(Tp^2Et^)_2_

EuI_2_ (0.406 g, 1.00 mmol), KTp^2Et^ (0.880 g, 2.09 mmol) were added to a 100 mL round-bottomed flask with 50 mL tetrahydrofuran in a glove box. The mixture was stirred for 12 hours at room temperature and then the solvent was removed. Pure product was obtained as crystalline powder after sublimation at 180 °C (0.460 g, 0.503 mmol, 50%). Anal. calcd for C_42_H_68_B_2_N_12_Eu: C 55.15, H 7.49, N 18.38; found: C 55.07, H 7.53, N 18.78.

## Supplementary information

Supporting Information

## Data Availability

[CCDC 2195888 (for Ce-TBO^2Et^) and 2195890 (for Eu(Tp^2Et^)_2_) contain the supplementary crystallographic data for this paper. These data can be obtained free of charge from The Cambridge Crystallographic Data Centre via www.ccdc.cam.ac.uk/data_request/cif.] The data that support the findings of this study are available from the corresponding author upon reasonable request.
