# Supplementary material for: Lanthanide complexes with d-f transition: new emitters for single-emitting-layer white organic light-emitting diodes
Source: Light Sci Appl. 2023 Jul 7;12:170. doi: 10.1038/s41377-023-01211-5 (PMC10328933; doi:10.1038/s41377-023-01211-5)
Supplement: Supplementary file 1 — Supporting Information [file 41377_2023_1211_MOESM1_ESM.pdf]

Supplementary Information for

Lanthanide complexes with d-f transition: New emitters for single-emitting-layer white organic light-emitting diodes

*Peiyu Fang, Peihao Huo, Liding Wang, Zifeng Zhao, Gang Yu, Yanyi Huang, Zuqiang Bian, Zhiwei Liu**

Beijing National Laboratory for Molecular Sciences, State Key Laboratory of Rare Earth Materials Chemistry and Applications, College of Chemistry and Molecular Engineering, Peking University

Beijing, 100871, P. R. China

P. Fang and P. Huo contributed equally to this work.

*Corresponding author. Email: zwliu@pku.edu.cn

1. Crystallographic Information

Table S1. Crystallographic data for complexes Ce-TBO^{2Et} and Eu(Tp^{2Et})$_2$.

Complex	**Ce-TBO^{2Et}**	**Eu(Tp^{2Et})$_2$**
Formula	$C_{70}H_{114}B_4N_{20}O_2Ce_2$	$C_{42}H_{68}B_2N_{12}Eu$
Fw	1591.29	914.67
Crystal system	Monoclinic	Monoclinic
Space group	*C 2/c*	*C 2/m*
a (Å)	22.8442(4)	17.2884(6)
b (Å)	14.2076(2)	12.7091(3)
c (Å)	25.9275(5)	11.3278(3)
α (°)	90	90
β (°)	113.029(2)	112.974(3)
γ (°)	90	90
V (Å^3)	7744.4(3)	2291.53(12)
F(000)	3304	954
Z	4	2
D_c (g cm^{-3})	1.365	1.326
μ (mm^{-1})	1.217	1.412
θ range (°)	2.684–30.654	2.051–29.364
Ref. meas./indep.	46391/10693	14516/3093
Obs. ref.[$I > 2\sigma(I)$]	8508	3077
R_{int}	0.0339	0.0331
R_1 [$I \geq 2\sigma(I)$]^a	0.0293	0.0274
ωR_2(all data)^b	0.0715	0.0652
Goof	1.026	1.018
$\Delta\rho$(max, min) (e Å^{-3})	0.458, -0.418	0.892, -0.597

$^a R_1 = \Sigma||F_o| - |F_c||/\Sigma|F_o|$. $^b wR_2 = [\Sigma w(|F_o^2|-|F_c^2|)^2/\Sigma w(|F_o^2|)^2]^{1/2}$.

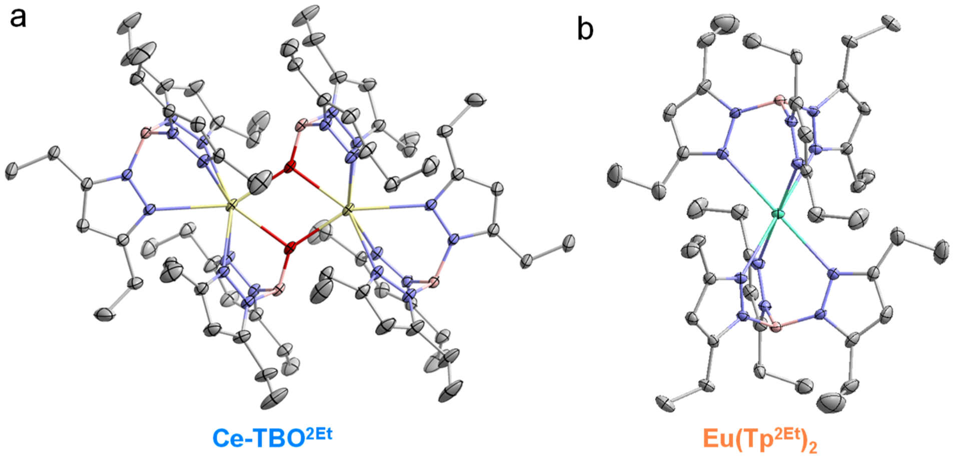

Figure S1. (a) ORTEP drawing of Ce-TBO^{2Et}. (b) ORTEP drawing of Eu(Tp^{2Et})$_2$. All hydrogens are omitted for clarification. Atom notation: Ce, yellow; Eu, cyan; C, gray; N, blue; O, red; B, pink.

2. Photophysical Properties and Theoretical Calculations

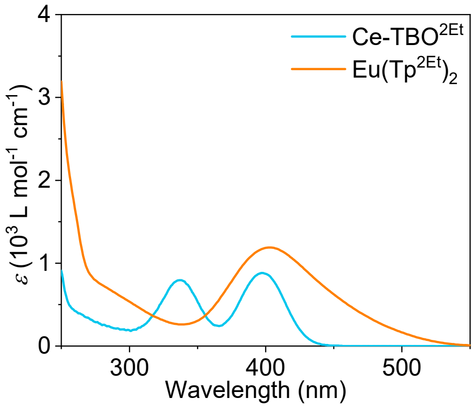

Figure S2. UV-Vis absorption spectra of Ce-TBO^{2Et} and Eu(Tp^{2Et})$_2$ in 1 mM dichloromethane solution.

Gaussian 16 Rev. C.01 was used for all electronic structure calculations.[1] The PBE0 hybrid density functional theory (DFT) method was employed, with a 28-electron small core pseudopotential on Eu and 47-electron large core pseudopotential on Ce with MWB28 or MWB47 basis separately, and the 6-31G* basis set for all other atoms. The optimized structures are used for the single point calculation and time-dependent

density functional theory (TD-DFT) calculation, and 28-electron small core pseudopotential and MWB28 basis are used for both Ce and Eu. Hole-electron analysis [2] and natural transition orbitals (NTOs) were calculated using Multiwfn 3.8.[3] Corresponding donor and acceptor orbitals from NTOs calculation were rendered with the program visual molecular dynamics (VMD).[4]

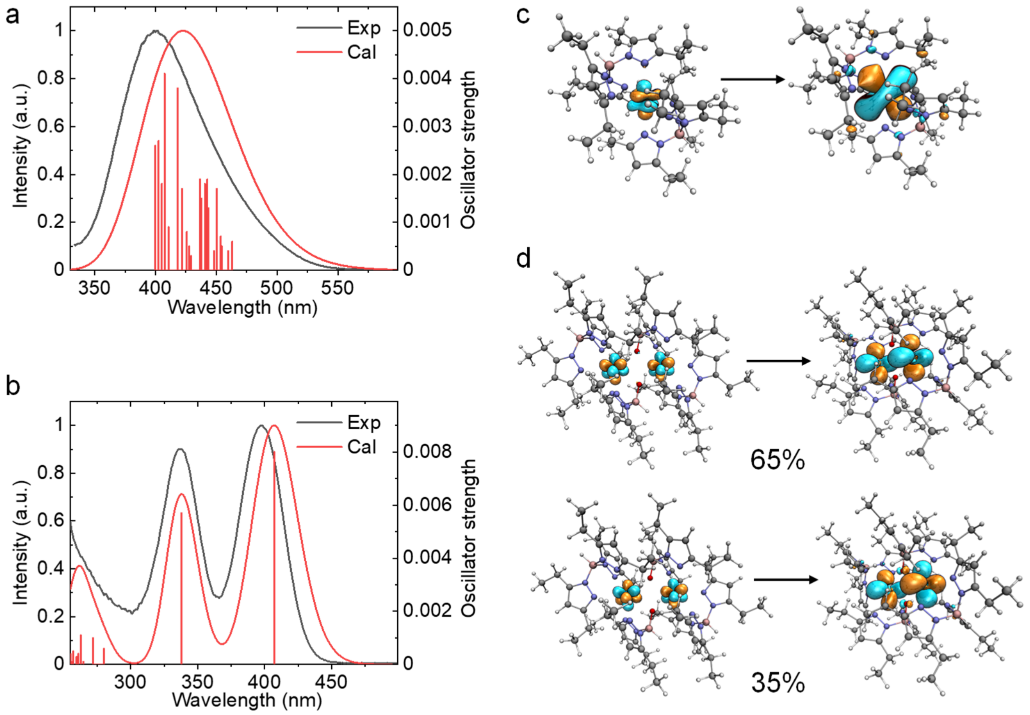

Figure S3. Experimental (black solid line) and TD-DFT calculated (red solid line) absorption spectra of (a) Ce-TBO^{2Et} and (b) Eu(Tp^{2Et})$_2$. The predicted spectra were rendered as Gaussian line shapes having a FWHM of 0.3 and 0.5 eV, respectively. Oscillator strengths for the electronic transitions are shown as red vertical lines. Calculated NTOs of the lowest energy transition for (c) Ce-TBO^{2Et} and (d) Eu(Tp^{2Et})$_2$ in gas phase with iso-surface value ±0.04. The transition characteristics of Ce-TBO^{2Et} are described by two pairs of NTOs, and the proportions are 65% and 35%, respectively.

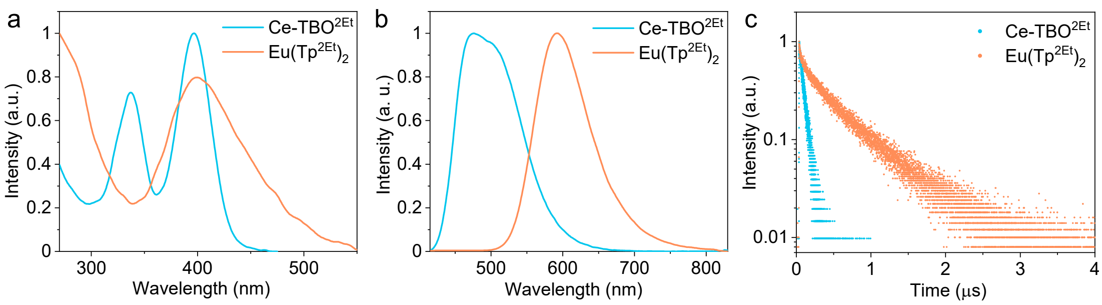

Figure S4. (a) Absorption spectra (b) Emission spectra and (c) transient photoluminescence decay curves of Ce-TBO^{2Et} or Eu(Tp^{2Et})$_2$ doped in PMMA film (10 wt%).

3. Thermal Properties

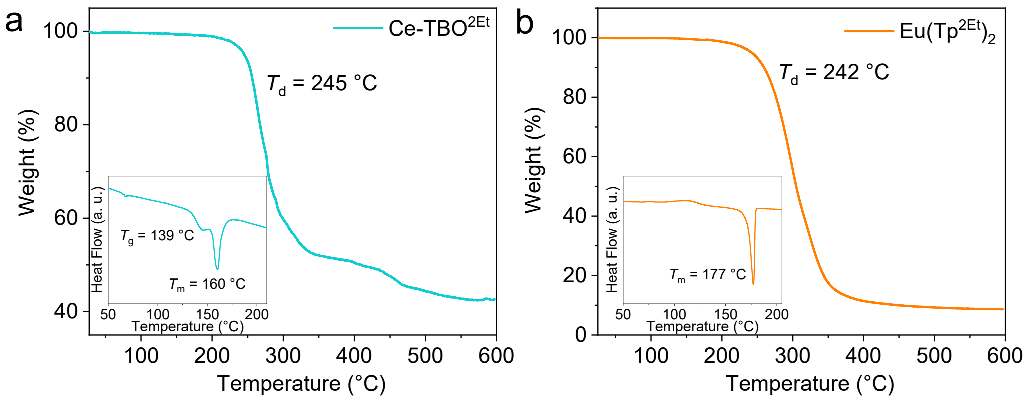

Figure S5. Thermal gravimetric analysis (TGA) inset with the differential scanning calorimetry (DSC) curve of (a) Ce-TBO^{2Et} and (b) Eu(Tp^{2Et})$_2$. T_d represents the temperature at 5% weight loss. T_g represents the glass transition temperature. T_m represents the melting temperature.

4. Electrochemical Properties

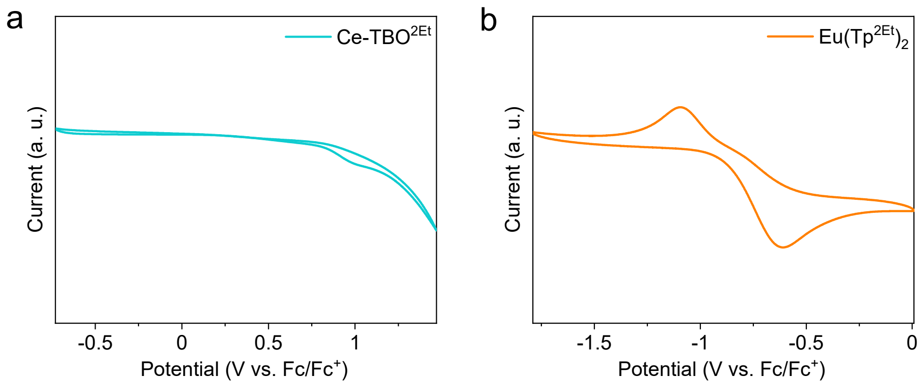

Figure S6. Cyclic voltammogram of (a) Ce-TBO^{2Et} and (b) Eu(Tp^{2Et})$_2$.

5. Electroluminescence Studies

Table S2. Photophysical data of Ce-TBO^{2Et} and Eu(Tp^{2Et})$_2$ doped films.

Host:dopant	$\lambda_{abs}{}^{a}$/nm	$\lambda_{em}{}^{b}$/nm	$\Phi_{PL}{}^{c}$/%
mCP:Ce-TBO^{2Et} (10 wt%)	270	470	88
	400	476	~100
NPB:Eu(Tp^{2Et})$_2$ (5 wt%)	300	594	46
m-MTDATA:Eu(Tp^{2Et})$_2$ (5 wt%)	300	592	46
mCP:Eu(Tp^{2Et})$_2$ (5 wt%)	300	591	57
	400	600	54

[a]Excitation wavelength, the shorter wavelength (270 or 300 nm) is used to excite host material, while the longer wavelength (400 nm) is used to excite the complex directly. [b]Maximum emission wavelength. [c]PLQY.

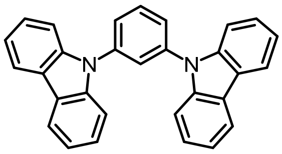

mCP

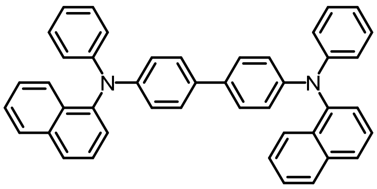

NPB

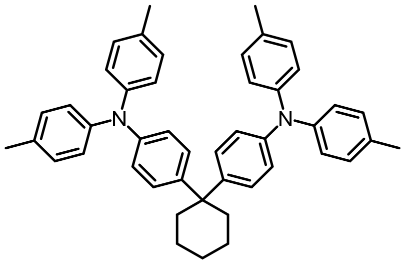

TAPC

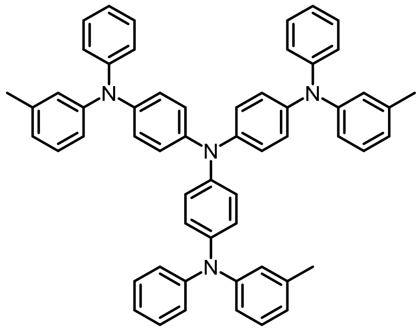

m-MTDATA

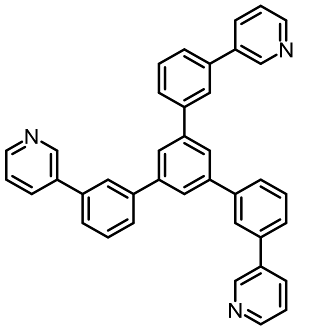

TmPyPB

Figure S7. The chemical structures of organic materials used in OLEDs study.

Table S3. Electroluminescent performance of reported OLEDs based on d-f transition lanthanide complexes.

Emitter	λ_{EL}[a]/nm	CIE	EQE_{max}[b]/%	L_{max}[c]/cd m^{-2}	Ref.
Ce-DC-18·C·6	382	-	-	-	5
$[Ce(triEtNTB)_2](CF_3SO_3)_3$	~460	0.18, 0.21	-	-	6
$Ce(pip)_3$	~500	-	-	-	7
Ce-1	434	0.15, 0.08	14.0	1008	8
Ce-2	472	0.17, 0.33	20.8	31160	9
3-Me	475	0.15, 0.23	14.1	33160	10
4-^{i}Pr	478	0.16, 0.25	1.1	1229	10
4-Pz	446	0.16, 0.15	0.55	108	10
$Ce(Tp^{Me2})_2(dtfpz)$	465	0.16, 0.21	1.4	1258	11
$Ce(Tp^{Me2})_2(dmpz)$	467	0.15, 0.24	9.9	36260	11
$Ce(Tp^{Me2})_2(dppz)$	482	0.19, 0.38	12.5	29200	11
Ce-TBO^{2Et}	587	0.18, 0.32	22.3	18200	This work
$Tp^*{}_2Eu$	596	0.54, 0.45	0.01	>10	12
Eu-1	510	0.14, 0.26	0.75	23	13
Eu-2	590	0.55, 0.44	6.5	30620	13
$EuBr_2$-N_8	520	0.26, 0.61	15.5	10200	14
EuI_2-N_8	520	0.36, 0.58	17.7	25470	14
$Eu(Tp^{2Et})_2$	588	0.54, 0.46	11.1	15800	This work

[a]Electroluminescence wavelength. [b]Maximum EQE. [c]Maximum luminance.

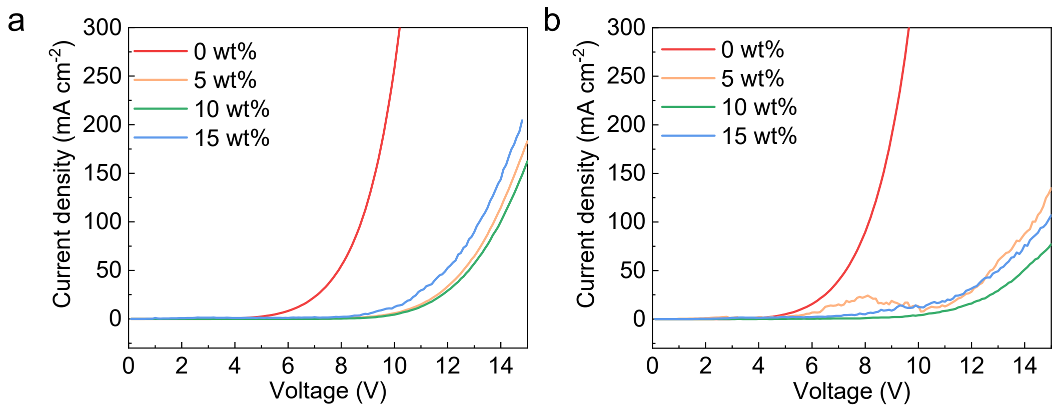

Figure S8. The current density-voltage curves of hole-only devices with different weight doping ratios of (a) Ce-TBO^{2Et} or (b) Eu(Tp^{2Et})$_2$ in mCP.

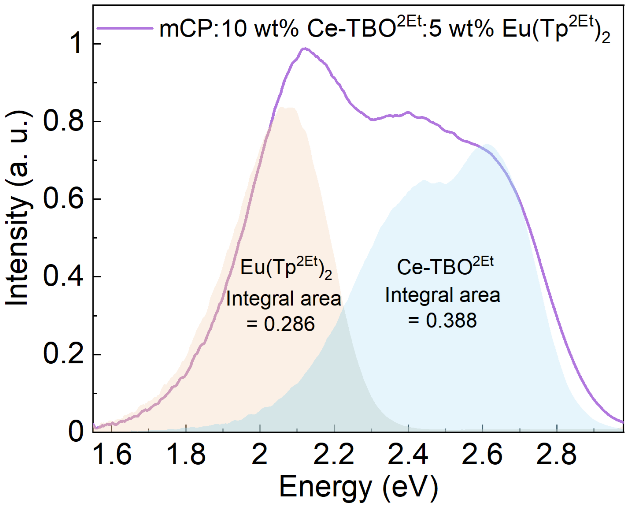

Figure S9. The peak splitting fitting of the emission spectrum of the co-doped film mCP:10 wt% Ce-TBO^{2Et}:5 wt% Eu(Tp^{2Et})$_2$ according to the emission peaks of Ce-TBO^{2Et} and Eu(Tp^{2Et})$_2$.

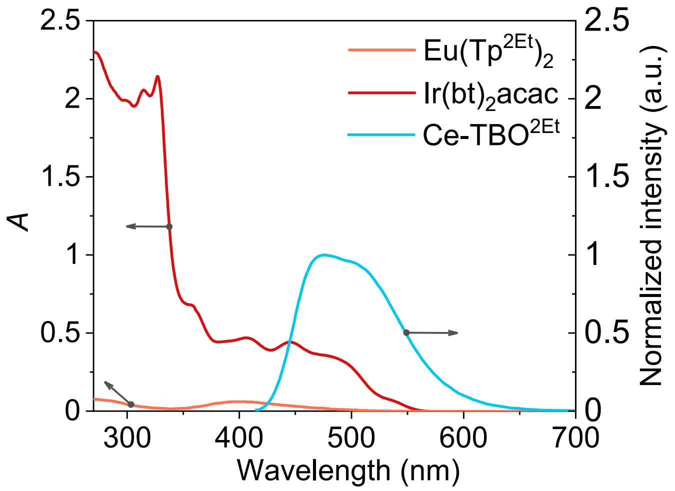

Figure S10. The absorption spectrum of 10 wt% Eu(Tp^{2Et})$_2$ or Ir(bt)$_2$acac doped PMMA film and the normalized emission spectrum of 10 wt% Ce-TBO^{2Et} doped PMMA film.

References

1 Frisch, M. J. *et al.* Gaussian 16, Revision C.01, Gaussian, Inc., Wallingford CT (2019).

2 Liu, Z. Y., Lu, T. & Chen, Q. X. An sp-hybridized all-carboatomic ring, cyclo[18]carbon: Electronic structure, electronic spectrum, and optical nonlinearity. *Carbon* **165**, 461–467 (2020).

3 Lu, T. & Chen, F. W. Multiwfn: A multifunctional wavefunction analyzer. *Journal of Computational Chemistry* **33**, 580–592 (2012).

4 Humphrey, W., Dalke, A. & Schulten, K. VMD: Visual molecular dynamics. *Journal of Molecular Graphics* **14**, 33–38 (1996).

5 Yu, T. Z. *et al.* Ultraviolet electroluminescence from organic light-emitting diode with cerium(III)–crown ether complex. *Solid-State Electronics* **51**, 894–899 (2007).

6 Zheng, X. L. *et al.* Bright blue-emitting Ce^{3+} complexes with encapsulating polybenzimidazole tripodal ligands as potential electroluminescent devices. *Angewandte Chemie International Edition* **46**, 7399–7403 (2007).

7 Katkova, M. A. *et al.* Lanthanide imidodiphosphinate complexes: Synthesis,

structure and new aspects of electroluminescent properties. *Synthetic Metals* **159**, 1398–1402 (2009).

8 Wang, L. D. *et al.* Deep-blue organic light-emitting diodes based on a doublet d–f transition cerium(III) complex with 100% exciton utilization efficiency. *Light: Science & Applications* **9**, 157 (2020).

9 Zhao, Z. F. *et al.* Efficient rare earth cerium(III) complex with nanosecond d−f emission for blue organic light-emitting diodes. *National Science Review* **8**, nwaa193 (2020).

10 Fang, P. Y. *et al.* Lanthanide cerium(III) tris(pyrazolyl)borate complexes: Efficient blue emitters for doublet organic light-emitting diodes. *ACS Applied Materials & Interfaces* **13**, 45686–45695 (2021).

11 Yan, W. C. *et al.* Highly efficient heteroleptic cerium(III) complexes with a substituted pyrazole ancillary ligand and their application in blue organic light-emitting diodes. *Inorganic Chemistry* **60**, 18103–18111 (2021).

12 Shipley, C. P. *et al.* Orange electroluminescence from a divalent europium complex. *Advanced Materials* **11**, 533–536 (1999).

13 Zhan, G. *et al.* Highly efficient and air-stable lanthanide Eu^{II} complex: new emitter in organic light emitting diodes. *Angewandte Chemie International Edition* **59**, 19011–19015 (2020).

14 Li, J. Y. *et al.* Highly efficient and air-stable Eu(II)-containing azacryptates ready for organic light-emitting diodes. *Nature Communications* **11**, 5218 (2020).
